# Understanding the Normativity of Health Technology Assessment: Ontological, Moral, and Epistemological Commitments

**DOI:** 10.1007/s10728-024-00487-x

**Published:** 2024-06-17

**Authors:** Bart Bloemen, Wija Oortwijn, Gert Jan van der Wilt

**Affiliations:** Department IQ Health Science, Radboud University Medical Center, Nijmegen, The Netherlands

**Keywords:** Health technology assessment, Normativity, Value judgments, Commitments, Non-invasive prenatal testing

## Abstract

The inherent normativity of HTA can be conceptualized as a result of normative commitments, a concept that we further specify to encompass moral, epistemological and ontological commitments at play in the practice of HTA. Based on examples from literature, and an analysis of the example of assessing Non-Invasive Prenatal Testing (NIPT), we will show that inevitable normative decisions in conducting an assessment *commits* the HTA practitioner to moral (regarding what makes a health technology desirable), ontological (regarding which effects of health technology are conceivable), and epistemological (regarding how to obtain reliable information about health technology) norms. This highlights and supports the need for integrating normative analysis and stakeholder participation, providing guidance to HTA practitioners when making normative choices. This will foster a shared understanding between those who conduct, use, or are impacted by assessments regarding what are conceivable and desirable outcomes of using health technology, and how to collect reliable information to assess whether these outcomes are (going to be) realized. It also provides more insight into the implications of different normative choices.

## Introduction

Health Technology Assessment (HTA) *informs* decision-making on health technology *to promote* an equitable, efficient, and high-quality health system [1]. Hence, HTA claims to *improve* decision-making *by providing and assessing information* on (un)intended consequences of health technology. This aim makes HTA inherently normative [2–4]. This normativity of HTA has been described before, and HTA practitioners (i.e. those responsible for conducting assessments, comprising scoping the research question; collecting, synthesizing and appraising evidence; and reporting findings and implications) increasingly acknowledge that their practice has normative aspects [2, 5].

Despite widespread recognition of HTA’s normativity, the adoption of approaches to address it, such as integrating ethical analysis to clarify value judgments made in HTA, remains rather limited [6]. A recent paper by Charlton et al. suggests that this results from a lack of exactness and consistency in the language used to articulate HTA’s normative aspects [4]. According to the authors, this leads to ambiguities in approaches to address normativity, undermining their ability to make normative reasonings explicit and scrutinize substantive rationales underlying HTA. Therefore, they propose a conceptual framework for articulating normative aspects of HTA.

We agree with Charlton et al. that HTA is inherently normative, that this normativity is often left implicit or underspecified, and that addressing this can enhance legitimacy of HTA. We propose to extend their framework by explicating their concept of *normative commitments*, further specifying it to encompass different types of normativity.

In this paper, we analyze different examples from literature, and provide an in-dept analysis of Non-Invasive Prenatal Testing (NIPT) to illustrate that the normativity of HTA can be understood as resulting from commitments regarding what are *conceivable* and *desirable* outcomes of using a health technology, and *how to gather reliable information* to establish whether these outcomes are (going to be) realized. These normative commitments guide HTA practitioners in making methodological decisions, *committing* them to underlying *moral, epistemological*, and *ontological* norms. Before presenting our analysis of normative commitments, we will briefly describe the complexity and importance of explicating normative aspects of HTA.

## Making the Invisible Visible 1

### …By Having a Language that Describes Normative Aspects of HTA

Normative aspects of HTA are often unarticulated and therefore hidden for all involved stakeholders [4]. HTA practitioners employ (in)formal norms that specify abstract values worth pursuing, in measuring and evaluating health technology’s impact. While guidelines may codify these norms, individual cases may require re-specifications or difficult trade-offs. Moreover, the relation between norms and their underlying values is often left implicit, obscuring their normative nature. It would enhance the legitimacy of HTA if this *invisible* normativity would be made *visible*. We agree with Charlton et al. that a clear language is needed to explicate this normativity, and propose to specify their concept of *normative commitments* to show that it encompasses various types of normativity in HTA and acknowledge their complex interrelations [4].

### …That Acknowledges the Entanglement of Norms and Evidence

Charlton et al. argue that judgements about what is valuable (‘*ethical judgments’*) operate *alongside* judgments about what one ought to believe given the available information (‘*evidential judgments’*). According to them, judgments on meaning and quality of evidence do not *determine* recommendations and decisions. Despite this being true in principle, evidential judgments, such as concluding whether a drug is effective based on available evidence, may have important implications that HTA bodies and/or decision-makers may be required to consider when making recommendations or decisions [7–9].

Charlton et al. also state that evidential judgements incorporate normative commitments which can ground knowledge claims [4]. By noticing this, they acknowledge a certain entanglement of norms and evidence in HTA. However, norms do not only play a role in evidence interpretation and judgements on its validity (e.g., thresholds for statistical significance), but also in evidence selection (what is considered evidence) and evidence generation (e.g., outcome measures used in HTA incorporate values by how they are measured) [10].

This entanglement of norms and evidence is not uncommon in HTA. When evaluating the effectiveness of health technology an implicit connection is made between a descriptive statement (e.g., ‘this drug will lower blood pressure’) and an evaluative premise (e.g., ‘by lowering blood pressure the risk of developing cardiovascular disease will be reduced, and this is a good thing to do’) [2, 11, 12]. HTA evaluates such mixed claims because it relies on concepts and classifications that are descriptive *and* evaluative. For example, what is considered a disease is based on descriptions of symptoms and an interpretation of these symptoms as something that requires (medical) treatment. Another example is that measuring *health-related quality of life* (HRQoL) requires empirical information on impact of a condition on aspects of life held to be valuable [2, 13]. The connection between a descriptive and evaluative premise may not be obvious, may also not be intended by HTA practitioners, but it should be there to inform decision-makers on whether a health technology is a ‘better’ option than alternatives [3, 12]. Consequently, the relation between evidence and norms in HTA is not one of independency but of entanglement [2, 13, 14].

### …And Acknowledges Normative Features of Health Technology

Moreover, social science has shown that health technology is not neutral. Using health technology often requires changes in how we do or organize healthcare practice; shapes the way we treat and think about health and disease; and creates new situations that challenge or demand rethinking of existing norms [15]. These normative features of health technology should be accounted for in HTA [16–18]. For example, when assessing the *effectiveness* of a health technology, it is important to consider that its ability to realize the intended effect depends on the context in which it is applied, but also that the intended effect itself could be different by re-designing the technology to realize another (valuable) effect. Because there may be disagreement about the desirable effect, assessing a given configuration of a technology may lead to assessing a health technology on effects deemed undesirable by some stakeholders. Therefore, HTA also needs to conceptualize *how* certain outcomes *can be realized* by using a health technology. These *ontological assumptions* are also part of the (implicit) normativity of HTA 2.

### …And Reflects the Role of HTA in Decision-Making

Finally, because of the role of HTA in decision-making, it can be used to prioritize (a particular use of) certain health technologies [15]. Therefore, HTA is an actor in decision-making, and its practitioners have a responsibility in providing justifications for this role. It can certainly not be expected from HTA practitioners alone to identify and justify all normative choices guiding HTA, but by being part of its practice they are *committed* to underlying rationales.

Considering this broader understanding of the normativity of HTA, making it visible is one aspect, but it is also important to acknowledge that normative commitments are *jointly produced in assessments* [19]. Therefore, a language that helps anyone involved in recognizing and explicating this normativity would be helpful, but it should encompass *all types of normativity* involved, their interrelatedness, and its actual use needs a consideration of *who is involved*.

### Normative Commitments in HTA

We define normative commitments as 3: “*any commitment to a norm that guides, and is further specified in, the assessment of information about the properties, effects or impacts of health technology. Different types of norms jointly provide justifications for conclusions about whether, and how, health technologies should be used. Commitments to these norms can be implicit but are shown by a willingness to deploy and defend them in justifying the rationales underlying an assessment*”.

Our concept of normative commitments highlights that every assessment is an expression of normative commitments of those involved in the process. There are three types of normative commitments, regarding how the world ought to be (*moral commitment*), which role health technology could play in realizing this (*ontological commitment*), and which information reliably indicates that intended outcomes are (going to be) realized (*epistemological commitment*). We will further define and illustrate these types of normative commitments and their implications for HTA by providing examples from literature. We start with a brief description of moral commitments and provide more extensive descriptions of epistemological and ontological commitments because moral commitments are already extensively discussed in literature on normativity of HTA [2–4]. Although the different types of normative commitments are introduced separately, in practice there will be many dependencies among them as discussed in the next section.

### Moral Commitments in HTA

Moral commitments are “*commitments to norms concerning what is desirable and acceptable*”. In HTA, these norms guide which effects of health technology are considered important based on commitments to values such as avoiding harm or promoting well-being, and norms concerning what constitutes legitimate decision-making (e.g., transparent and inclusive processes).

In HTA, not all conceivable effects of health technology are assessed, the focus is on consequences that matter to us. Moral considerations guide distinctions between beneficial and harmful consequences to assess desirable qualities of health technology [3]. For example, cost-effectiveness analyses often use quality-adjusted life years (QALYs) to measure benefits which entails a commitment to maximizing HRQoL for recipients of care. However, alternative conceptions of the good that include broader aspects of well-being (beyond health) or benefits beyond recipients of care may also inform such analyses [22–24].

## Epistemological Commitments in HTA

Epistemological commitments are “*commitments to norms concerning the nature of knowledge, how it can be acquired, and how its reliability can be evaluated and justified*”. In HTA, these norms define which types of information can support conclusions on effects of health technology, based on commitments to theories about what is reliable knowledge. Where moral commitments guide what we consider desirable consequences of health technology, epistemological commitments concern what can be reliably stated about these consequences. For example, although a shared moral commitment to maximizing HRQoL can lead us to consider the QALY as outcome measure, diverging epistemological commitments can lead to different ideas about how QALYs should be estimated (e.g., is a patient a reliable judge on his or her quality of life? Can quality of life be reliably quantified?).

Important normative epistemological questions in HTA concern: *who decides what forms of information count as appropriate evidence in HTA*, and *who is recognized as a credible source of knowledge or information* [25, 26]. Although the epistemology of HTA (aligned with principles of evidence-based medicine) does not exclude consideration of (qualitative) experiential knowledge, HTA agencies prefer quantitative types of evidence [25–27]. Also, when assessing impact on quality of life, outcome measures constructed without patient involvement are used [28].

The link between who is involved in evidence-making processes and fair decision-making has drawn recent attention in discussions about evidence use in HTA [26, 29]. HTA agencies have also been challenged, even taken to court, regarding their interpretation and use of evidence, mainly taking from RCTs. For example, the Dutch National Health Care Institute was sued for misconduct against interstitial cystitis patients because, according to the patient’s association, it attributed *too little credibility* to the experience of doctors and patients concerning potential effects of bladder instillations [30]. The exclusion of experiences and perspectives as an (unintended) result of epistemic norms upheld by HTA agencies is also reported in studies that evaluated stakeholder participation at HTA agencies. Because experiential knowledge contributed by patients and clinicians does not fit prevailing ideas of robust evidence, their ability to engage in discussions on HTA outcomes is limited [31–33].

The role of epistemological commitments in HTA is also evident in discussions on the use of real-world data (RWD). Despite scholars highlighting inherent limitations of RCTs such as generalizability to real-life clinical contexts, and situations in which RCTs are difficult to conduct (e.g., due to small population sizes or characteristics of a technology), the validity of RWD is debated [34]. Even when consideration of RWD in assessments is required by law, some HTA practitioners assign a low credibility to it (effectively excluding it from impacting recommendations) [34]. However, in some cases (e.g., orphan diseases, end-of-life treatments) HTA agencies and practitioners are willing to consider other types of evidence or thresholds, based on overriding moral commitments [35].

## Ontological Commitments in HTA

Ontological commitments are “*commitments to norms regarding the nature of reality, the existence of certain entities or phenomena, and plausible causal mechanisms*”. In HTA, these are ideas about which types of interventions could impact health, and which effects are conceivable, based on theories about mechanisms of health and disease, the working mechanisms of technology, and the organization of healthcare. Where moral commitments concern what are desirable consequences of health technology, and epistemological commitments guide ways of finding out whether these consequences do happen, ontological commitments concern what could happen. For example, when estimating QALYs it is important to consider what constitutes HRQoL and identify aspects changeable by technology. This involves defining the nature of health (e.g., from a view that health is the absence of disease a health technology can improve HRQoL by eliminating symptoms and disabilities, whereas from a conception of health as the ability to experience a state of well-being a health technology can also improve HRQoL by improving social relations or living conditions) [11, 24].

An implicit ontology also guides the organization of HTA processes and the conduct of assessments.

Firstly, the decision problem in HTA is framed as a question concerning the use of a health technology. This is reflected in the assigned remit of HTA agencies that is often linked to a single class of technologies (e.g., drugs, medical devices), and in the scope of assessments (e.g., relative effectiveness of a *single* technology compared to a standard of care) [9, 36]. Evaluating health technologies individually may neglect undesirable cumulative effects, such as shifts in societal norms regarding responsibility for one’s own health due to the omnipresence of health checks (e.g., screening programs, diagnostic tests) [37].

Secondly, outcome measures used in HTA, like the QALY, embed ontological presumptions. Measuring QALYs relies on questionnaires by which patients can report the impact of their health condition on aspects of life deemed important for evaluating *quality of life*. This not only implies a moral commitment to which aspects of life are desirable, but also what *constitutes* quality of life. Although there are theories that focus on other constituents of quality of life, HTA has predominantly relied on a utilitarian conception [38]. The use of instruments that are based on different conceptualizations (e.g., the capability approach) may lead to different assessments and priorities in healthcare [39].

Finally, health technology may imply something about the nature of a health problem. Increasingly, technologies are used in diagnosing and treating people, distinguishing between healthy and unhealthy, making visible biological processes and functions held to be constitutive of a disease. These are not just ontological classifications, but also invoke ideas about what is normal and desirable. For example, cochlear implants imply, by aiming to restore normal hearing, that deafness is a disability that should be resolved; and interventions for autism suggest that its associated behavior is not part of normal functioning [40]. Assessing such technologies on intended effects, without questioning the underlying problem definition, risks evaluating them on outcomes that are unacceptable to certain stakeholders.

### The Interrelatedness of Normative Commitments in HTA

While our description above intentionally separated normative commitments for clarity, in practice they are interdependent and may align or be in conflict. For example, there is a broadly shared moral commitment to involve those that are potentially impacted by an HTA informed decision, creating tensions with epistemological commitments to only consider reliable evidence, excluding experiential knowledge as formal evidence. It is challenging to resolve these tensions. Realizing that the epistemology of HTA is grounded in moral commitments (i.e., objective knowledge is needed to evaluate the *public* value of health technology) may be a constructive way forward [41]. It acknowledges that *epistemic acts* (generating and interpreting evidence) do not merely capture aspects of reality but also shape reality [42].

Establishing an a priori hierarchy for balancing commitments is challenging, as commitments need case-specific specifications. However, articulating normative commitments during an assessment can reveal their connections and facilitate coherence.

An example of how articulating different commitments can facilitate coherence is an HTA conducted by the Finish Council for Choices in Health Care (COHERE) [43]. Staff of COHERE was asked to conduct an assessment to give recommendations to the Ministry of Social Affairs and Health about public funding of medical treatments for gender dysphoria. Their approach was to integrate ethical analysis in every step of the assessment (scoping, evidence gathering, and appraisal), by embedding ethicists in a multidisciplinary HTA team and organizing stakeholder hearings. This led to an identification of different normative issues and revealed relations between different commitments:

- Moral commitments to autonomy (people should be able to decide a gender identity themselves) are related to epistemological commitments regarding high quality of evidence because autonomous decision making requires having reliable information on life-long consequences of choices.
- Ontological commitments to classifications of gender dysphoria (distinguishing between people with transgender identity, people with a nonbinary gender identity, and minors with variations in gender identity) potentially conflict with a moral commitment to equity (why should subgroups receive different treatment?).

Bringing these normative considerations together led to a recommendation for psychosocial counseling and being cautious about offering permanent treatments to adolescents (considering the uncertainty in predicting long-term outcomes which also impedes autonomy), and highlighted equity issues regarding the (currently) different treatment of people with transgender or non-binary gender identity.

We will further demonstrate the usefulness of our concept of normative commitments in explicating normative aspects in the assessment of NIPT.

### Example: Assessment of NIPT

In this example, HTA informs a decision on implementing NIPT in a national prenatal screening program. NIPT is a procedure that analyses fetal DNA in the mother’s blood to obtain information about the fetal genotype [44]. The procedure only requires a blood sample of the mother and does not pose risks of a miscarriage associated with other (invasive) prenatal tests (e.g., amniocentesis, chorionic villus sampling). It offers other potential advantages such as early testing (around 10th week of pregnancy) and more reliable and comprehensive information on genetic conditions of the unborn child.

Given NIPT’s diverse potential uses in prenatal screening, decisions are needed concerning its implementation, e.g. offer it commercially or within a national screening program, as a first line test or supplement to other prenatal tests, to all pregnant women or women with particular risks, with or without reimbursement by health insurance, and screening specific genetic conditions or providing whole genome coverage [44]. This multiple realizability of NIPT is mirrored by different views people may have on how NIPT should be used, which effects are (un)desirable, and how to evaluate whether these effects are (going to be) realized [45].

The HTA practitioner’s task is to demonstrate, by collecting, synthesizing, and appraising (quality of) evidence, the potential effects of different implementations of NIPT and how these compare with alternative prenatal screening options. Despite legal and methodological guidelines on how an assessment should be done, several decisions must be made.

Firstly, it should be decided whether NIPT needs an assessment and what the relevant comparators are, reflecting *prior moral and ontological commitments*. For example, the decision to assess NIPT classifies it as a *health* technology, useful in realizing *health-related* benefits. This classification is not unquestionable, as shown by critical responses that the HTA agency of Germany (the Federal Joint Committee, G-BA) received. Because the G-BA only has a mandate for assessing technologies with a *medical* purpose, their decision to assess it implied a purpose of NIPT that was contested by some stakeholders [46]. Moreover, using prenatal screening technologies such as NIPT can be seen as an expression of *disvalue* for people with disabilities [47]. It also raises ontological questions concerning whether the disabilities themselves make disabled people worse off or the societal conditions (e.g., prejudices and discrimination, lack of societal support), questioning the focus on technological solutions.

Secondly, scoping includes identifying outcomes to consider in the assessment which reflects *prior moral commitments* about what makes a health technology desirable [14, 48]. In the case of NIPT, its purpose may be seen as *providing information* on genetic abnormalities, or *prevent the birth of children* with genetic abnormalities, or *enhance reproductive autonomy* [45]. These ideas about the primary purpose of NIPT implicitly guide the assessment of its safety, clinical effectiveness, and cost-effectiveness. To assess effectiveness, decisions are necessary regarding which and whose benefits to consider (e.g., only the benefits for prospective parents or also for the unborn child, which genetic anomalies are important to detect) focusing on either its ability to effectively help prospective parents making decisions or to prepare a life for the unborn child. Assessing cost-effectiveness also leads to methodological issues that imply value judgments. For example, using QALYs as an outcome would imply, depending on whether maternal or fetal QALYs are considered, that NIPT is cost-effective to the extent that it prevents a sufficient number of births affected by genetic disability or helps supporting the lives of children with disabilities [45, 49].

Once the assessment scope is determined, evidence collection and appraisal occur. Decisions must be made, guided by *epistemological commitments*, on the types of information to include and to what extent they constitute *evidence of* NIPT achieving specific outcomes. This may result in the exclusion of conceivable and important outcomes (e.g., changing social norms and attitudes towards people with disabilities) due to the unavailability of information that meets certain methodological criteria to be counted as evidence.

**Fig. 1:**
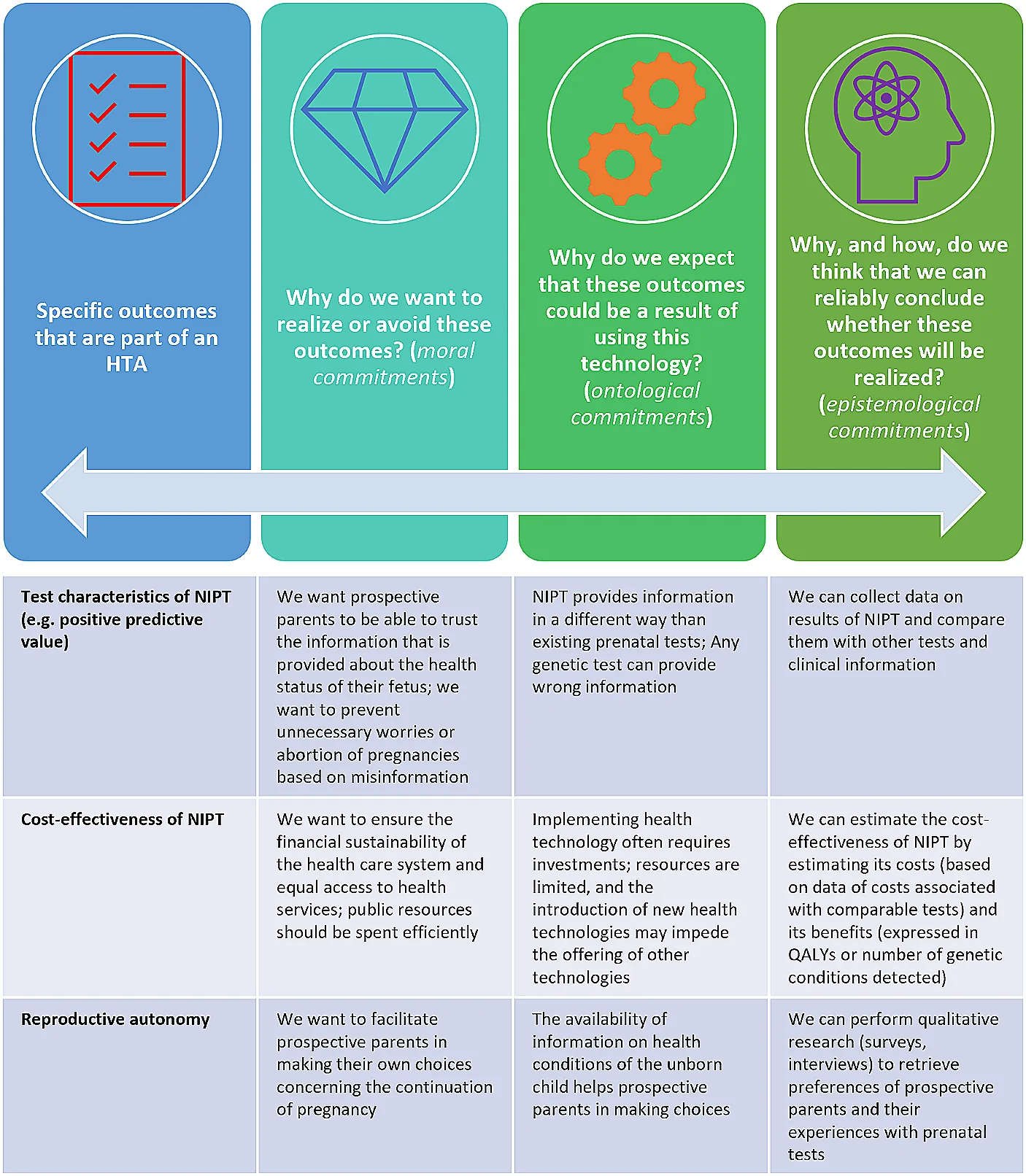
The role of normative commitments in assessing NIPT, grounding the use of particular outcomes

To conclude, our analysis highlights different ontological, moral, and epistemological commitments that jointly shape an assessment of NIPT, but also illustrates their close interrelatedness. Because HTA informs public decision-making, which can have moral consequences such as impacting availability of NIPT, a justification of using certain (even epistemological and ontological) norms will include references to moral ideas concerning justice and the good life (e.g., a particular operationalization of the QALY will partly be justified by reference to ideas about what makes this technology acceptable). Despite this interrelatedness among types of normative commitments, there are relevant differences. For example, although it is conceivable that NIPT leads to more terminations of pregnancies, and reliable data about this effect can be obtained, some HTA agencies have decided to ignore this as outcome and focus on enhancing reproductive autonomy because of overriding moral commitments. Therefore, a particular co-specification of normative commitments takes place in an assessment, which may look like the schematic example in Fig. 1 [13].

## Discussion

### Implications for the Practice of HTA

We have argued that the normativity of HTA is a result of moral, epistemological, and ontological normative commitments that guide its practice. These commitments are often left implicit but express themselves by what HTA practitioners do. What are the practical implications of this? How can we make these normative commitments more visible? As we will argue, this requires integrating normative analysis and stakeholder participation.

### Integrating Normative Analysis in HTA

**Fig. 2:**
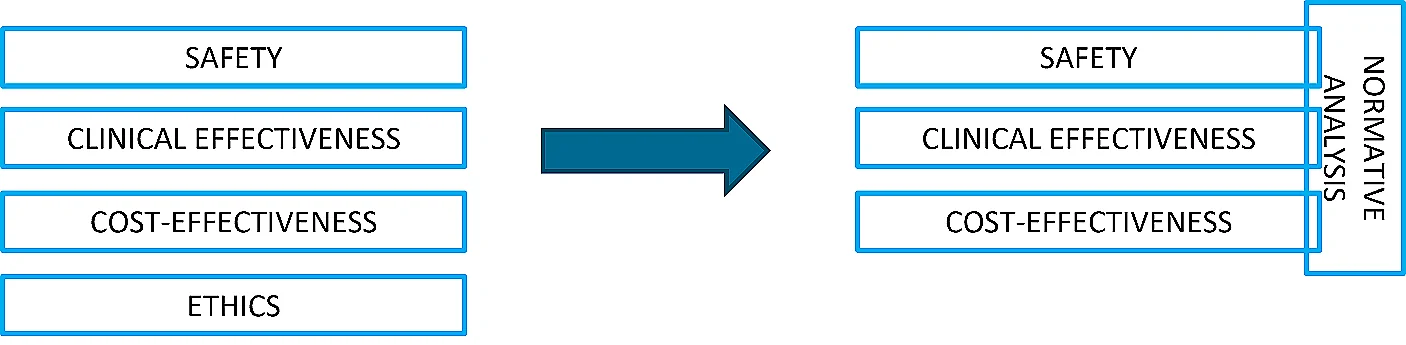
Instead of viewing normative analysis as a separate analysis conducted in HTA (e.g., ‘ethics’), it should be seen as an integral part of assessments. Adapted from [40]

Normative analysis should be integral to HTA, explicating and justifying the moral, epistemological, and ontological commitments underpinning the assessments, and explaining how they jointly produce a reliable, relevant, and coherent assessment of the potential value of a health technology (see Fig. 2). Traditionally, assessments are divided into evaluations of different ‘aspects’ of health technology, e.g., safety, effectiveness, cost-effectiveness, and ethical analyses. Although they emphasize different qualities or consequences of health technology, these distinctions disguise their dependencies (e.g., improving safety may require safety measures that make using a technology more time consuming, reducing cost-effectiveness) [48]. This division into different evaluations may also lead to conflicting results. For example, whereas ethical analysis may suggest that NIPT’s purpose should be to facilitate informed choice, cost-effectiveness analysis may use outcome measures (e.g., cost per additional chromosomal abnormality detected) that frame the purpose of NIPT as preventing a sufficient number of affected births (i.e., because that makes NIPT more ‘cost-effective’). Consequently, decision-makers using the outcomes of such HTA are confronted with different results that imply conflicting ideas about the desirable use of NIPT [45].

To enhance coherence of assessments, we should clarify the moral commitments underlying different evaluations. For example, the safety of health technology is evaluated *because we are committed* to the moral principle of doing no harm, and it requires *moral distinctions* between intended effects (which improve clinical effectiveness) and undesirable side effects (threatening safety) [14, 40]. Explicating these moral commitments aids in making connections between different analyses, as safety, clinical effectiveness, and costs-effectiveness all ascribe qualities to a health technology that make a discrete contribution to its (perceived) value [3, 48]. An articulation of how these different qualities contribute to ends that are being pursued by using that technology improves coherence between different analyses [50]. For example, normative analysis may show that NIPT is broadly valued for its ability to expand parental choices during the prenatal care pathway. Using outcome measures in safety, effectiveness and cost-effectiveness analysis aligned with this ability ensures coherence between these different analyses [45].

Additionally, the ontological and epistemological commitments underlying analyses should be clarified. Clarifying why specific effects of a health technology are evaluated involves moral ideas about what is relevant as well as background ideas about what is conceivable (i.e., which effects are, given known mechanisms underlying a disease and the workings of a technology, plausible), and about how to obtain reliable information about these effects.

Integrating and conducting this normative analysis in HTA requires embedding ethicists, with experience in health policy or HTA processes, in the HTA team. These experts could help in articulating the normative commitments at play [2, 43, 51].

### Stakeholder Participation in HTA

Because normative commitments influence *what* is assessed in HTA, and *how*, and impact public decision-making, an important question is *whose* commitments are explored in HTA. This foregrounds the importance of stakeholder participation in HTA to ensure that all relevant epistemological, ontological, and moral views are considered, and commitments are *jointly* produced. Because commitments are partly established during assessments, stakeholders should not just be consulted but participate in all phases of HTA processes. Moreover, given the often-implicit nature of normative commitments in HTA, the involvement of different perspectives may also help in making them visible. Therefore, someone’s voice may be relevant because he or she is affected by outcomes of an assessment, and/or because of the enlightenment and broadening of *perspectives* such voices provide [14]. The focus should not be primarily on *stakeholder* involvement as such, but on ensuring that all feasible and relevant *perspectives* are included in assessments [14].

Engaging stakeholders early in HTA processes could help determining the scope of assessments, clarifying what the specific health problem is that should be resolved by a health technology, identify alternative solutions and qualities that desirable solutions should have, and how it can be established whether desirable outcomes are (going to be) realized [40]. This should establish from which shared moral, ontological, and epistemological commitments the assessment can proceed.

During assessment, stakeholders can have a more substantial role besides providing comments. For example, in building models for cost-effectiveness analysis, stakeholders can provide normative guidance by co-deciding on what to include in the model and how to interpret its findings, which are normative choices [52]. Conducting cost-effectiveness analyses starting from different normative premises, for example with and without assuming equivalent value of QALYs (i.e., irrespective of characteristics of patients), may provide empirical data on the sensitivity of outcomes to different normative presumptions [53]. This can either lead to a conclusion that outcomes of the HTA are invariant to different normative premises, improving its robustness, or lead to more insight into what the normative disagreements are and their impact on conclusions of HTA.

### Responsibility and Accountability of HTA

Making normative commitments of HTA visible is a shared responsibility among all those who are involved in, or make use of, HTA. Although the normativity of HTA is shaped by contextual factors (e.g., available time, capacity, existing laws and guidelines), HTA practitioners can make important contributions to explicating and justifying this normativity. Integrating stakeholder participation and normative analysis into HTA processes provides practitioners with normative guidance, distributing the responsibility for justifying normative choices among all involved parties.

### Limitations

Our conceptualization of the normativity of HTA draws from literature and analysis of an example (NIPT) [13]. Because NIPT is a morally challenging health technology, the generalizability of our conclusions may be limited because we have identified issues specific for NIPT. Some of the normative aspects of HTA that we have highlighted may be less salient in other health technologies (e.g., drugs and medical devices) that are mostly assessed by HTA agencies. However, as health technology always has implications for the lives of people, assessing these implications will always touch upon normative issues, invoking judgments about which benefits represent societal value, as described previously in literature [2, 14, 15]. Also, in analyzing normative commitments we have drawn on literature describing normative aspects of HTAs of different types of technologies (including drugs). Therefore, we are confident that we have described normative aspects of HTA that have a generic nature.

We are aware that included literature may not fully represent the views of HTA practitioners, given also the highly contestable nature of concepts like ‘value judgment’, ‘objectivity’, ‘normativity’ etc., and different views about the nature and purpose of HTA, which requires further research and debate.

## Conclusion

We have argued that HTA is a normative practice, being an expression of ontological, moral, and epistemological commitments. Our specification of normative commitments provides a conceptual tool that could facilitate integration of normative analysis and stakeholder participation in HTA, bridging the gap between normative analysis and empirical inquiry. It also explains why stakeholder involvement is of intrinsic value to HTA, beyond its instrumental role in gaining acceptance. Moreover, it integrates insights from social science, philosophy of science and technology, concerning the complex interplay of technology, evidence, values, and science in evidence-based decision-making.

## Footnotes

1 This metaphor, which we think nicely describes the task at hand in addressing the normativity of HTA, was inspired by an interesting paper discussing the normativity of Technology Assessment (TA): Lucivero, F., Delvenne, P., & Van Oudheusden, M. (2019). Making the Invisible Visible. *TATuP*, *28*(1)

2 Ontology refers to the set of categories used to describe the nature of objects, their relations, and phenomena held to exist.

3 With ‘normative’ we refer to ideas about how things *should be* or how people should behave. These ideas can be formalized by norms and are expressed by normative judgments or acting in accordance with certain norms. In the context of HTA, ‘normative’ refers to ideas, norms, and standards that are tacitly understood within its practice as the right way to conduct assessments of health technology [20]. There are different types of norms that share a prescriptive and evaluative function, and moral norms (value judgments) are a subset of this [21]. For example, consider the difference between ‘this is a fair distribution of healthcare resources’ and ‘this body of evidence can be considered reliable’. Both express an evaluation, but the goal of the evaluation is different, i.e., one prescribes that you ought to consider a particular distribution of resources as morally acceptable, whereas the other prescribes that you ought to consider particular evidence as truthful. In HTA, because it informs public decision-making that has moral consequences, the justification of different norms always includes references to moral norms.

## Declarations

### Permissions

The authors have permission to use the content of the figures as they appear in the paper.
